# Synthetic Life with Alternative Nucleic Acids as Genetic Materials

**DOI:** 10.3390/molecules25153483

**Published:** 2020-07-31

**Authors:** Peng Nie, Yanfen Bai, Hui Mei

**Affiliations:** Shenzhen Key Laboratory of Synthetic Genomics, Guangdong Provincial Key Laboratory of Synthetic Genomics, CAS Key Laboratory of Quantitative Engineering Biology, Shenzhen Institute of Synthetic Biology, Shenzhen Institutes of Advanced Technology, Chinese Academy of Sciences, Shenzhen 518055, China; peng.nie@siat.ac.cn (P.N.); yf.bai@siat.ac.cn (Y.B.)

**Keywords:** synthetic biology, xenobiotic nucleic acids (XNAs), genetic material, synthetic life

## Abstract

DNA, the fundamental genetic polymer of all living organisms on Earth, can be chemically modified to embrace novel functions that do not exist in nature. The key chemical and structural parameters for genetic information storage, heredity, and evolution have been elucidated, and many xenobiotic nucleic acids (XNAs) with non-canonical structures are developed as alternative genetic materials in vitro. However, it is still particularly challenging to replace DNAs with XNAs in living cells. This review outlines some recent studies in which the storage and propagation of genetic information are achieved in vivo by expanding genetic systems with XNAs.

## 1. Introduction

**Figure 1:**
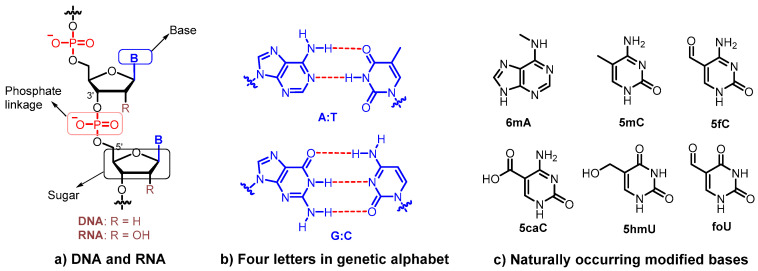
Structures of genetic materials. (**a**) Structures of DNA and RNA containing sugar, base, and phosphate moieties; (**b**) Watson–Crick base pairs formed by A:T, and G:C in DNA; (**c**) Examples of modified bases observed in nature.

DNA is the fundamental genetic material of all living organisms on Earth that stores and propagates genetic information. It is widely acknowledged that DNA, with a very uniform structure, is composed of four nucleobases (A, adenine; G, guanine; C, cytosine; T, thymidine), 2′-deoxyriboses, and charged phosphate backbones (Figure 1a). Four letters in the genetic alphabet form two base pairs (A:T and G:C) following the complementary rule, which is essential for the formation of a double helix structure and genetic information transmission (Figure 1b) [1].

Nevertheless, in nature, considerable chemical variations of the nucleobase, normally referred to as epigenetic markers, are observed in both prokaryotic and eukaryotic genomic DNA [2,3,4,5,6]. Modified bases, including but are not limited to N6-methyladenine (m6A), 5-methylcytosine (5mC), 5-formylcytosine (5fC), 5-carboxycytosine (5caC), 5-hydroxymethyl-uracil (5hmU), and 5-formyluracil (foU), have been identified to convey epigenetic information [5] (Figure 1c). In eukaryotes, such modifications function as a regulatory element, representing a second layer of regulatory information beyond the essential information encoded in the base sequence [4]. In mammals, 5mC and 5hmC are abundant in the brain and play an important role in organizing the chromatin structure and regulating gene expression [7]. Methylated nucleobases, such as m6A and 5mC, are observed in bacteria to protect cellular DNA from endonuclease-mediated cleavage that destroys the invading bacteriophage or viral DNA [8]. Moreover, m6A and 5mC are also active in cellular processes, such as DNA replication, transcription, transposition, and DNA repair [6]. The discovery of novel DNA modifications with different biological functionalities has become an attractive research area.

In most living organisms, only a minor portion of the nucleobases are modified in genomic DNA to bring in regulatory or protective functionalities. However, certain modified nucleobases can completely replace the canonical bases in some bacteriophages [6,9]. For example, in the DNAs of *Bacillus* phages SPO1, SP8, H1, 2C, and SP82, thymidine is completely replaced by 5hmdU [10]. Thymidine is substituted for deoxyuracil (dU) in the whole genome of *Bacillus* subtilis bacteriophages PBS1 and PBS2 [11,12]. Specific metabolic pathways are discovered in these phages that produce altered intracellular dNTP pools to synthesize modified DNA that are capable of escaping from host DNA repair systems, providing a possible method of using living cells with engineered genes that mimic phage genes for the incorporation of synthetic nucleotides into artificially built genetic systems [13,14].

To investigate the key chemical and structural parameters for genetic information storage, heredity, and evolution in vitro, a series of xenobiotic-nucleic acids (XNAs) are synthesized by replacing natural bases, sugars, and phosphate linkages with their unnatural counterpart. Modification of three subunits of nucleotides leads to sugar-modified XNAs, phosphate-modified XNAs, as well as base-modified XNAs, or their combination. Some of these XNAs can mimic natural nucleic acids to form a stable double helix between DNA/RNA or themselves following Watson–Crick base-pairing rules. The biological evaluation of these XNAs gives inspiring insights into the question of why nature chooses DNA/RNA as genetic materials rather than other chemicals, a fundamental question of the origin of life.

To further understand the potential of XNAs for genetic heredity, XNA replication should be accomplished. Genetic information transfer is achieved through DNA replication mediated by DNA polymerases in nature. However, in most cases, XNA building blocks, unnatural nucleotides, are not good substrates for natural DNA polymerases due to their high specificity [15,16]. Under such circumstances, laboratory-evolved XNA polymerases have been adopted to transmit and propagate the genetic information stored in XNAs with improved efficiency and fidelity [17,18]. Recent achievements proved that some XNAs function as alternative genetic materials in vitro with the function of information storage and propagation [19,20,21].

The following question is whether these XNAs can replace DNAs, and function as genetic materials inside living cells. Due to the complex biological and chemical environment, the invasion of XNA into a living cell is far more difficult to achieve. In this review, we will briefly introduce different kinds of XNAs with various chemical modifications and specific biological functions, and summarize some initial studies on the in vivo implementation of building xenobiotic life with XNAs. We hope that this review will encourage more systematic research on the exploration of XNA-based synthetic biology.

## 2. XNAs in Synthetic Biology

### 2.1. Sugar-Modified XNAs

**Figure 2:**
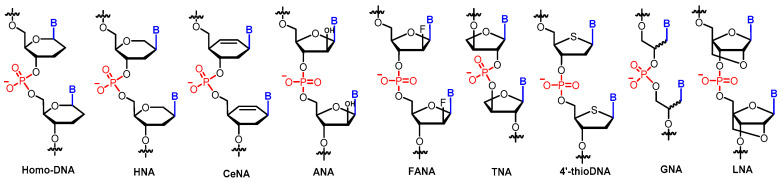
XNAs with artificial backbones.

One of the motivations to study XNAs is to develop modified oligonucleotides with improved chemical and biological properties, which can enable them to function more effectively over DNA/RNA for biomedical, biotechnology, and nanotechnology applications. Sugar-modified XNAs, such as bicyclo-DNA [22], LNA (locked nucleic acid) [23,24], and HNA (hexitol-nucleic acid) (Figure 2) [25], have been developed and display better chemical stability, superior nuclease resistance, and improved pharmacokinetic properties. Another goal is to explore possible explanations for why nature chooses ribofuranosyl sugar-based nucleic acids as information carriers. Thus, XNAs with “potentially natural” sugar moieties are developed and examined in the Eschenmoser group [26]. Sugars, including arabinose, lyxose, threose, allose, mannose, and glucose, are postulated to coexist with ribose under prebiotic circumstances.

Modified nucleic acids with various cyclic or acyclic sugar moieties were synthesized and systematically studied regarding their duplex formation between themselves or with DNA/RNA [26]. It was found that the stability of these sugar-modified XNA duplexes varies significantly. For example, the stabilities of allose-NA and altrose-NA duplexes are very low [26]. In contrast, homo-DNA, an oligonucleotide composed of 2′,3′-dideoxyallopyranose instead of 2′-deoxyribofuranose, forms a more stable Watson–Crick duplex than the corresponding DNA [27]. Interestingly, homo-DNA does not cross-pair with DNA or RNA as homo-DNA shows a ladder-like geometry but not the typical B/A-form helical geometry observed in DNA or RNA, indicating that homo-DNA is not able to transfer genetic information to DNA [27]. Only a small number of XNAs can form a stable antiparallel duplex with DNA/RNA following the canonical base-pairing rules, with examples including HNA [25,28], TNA (threose nucleic acid) [29], CeNA (cyclohexenyl nucleic acid) [30], ANA (arabino nucleic acid) [31], FANA (2′-fluoro-arabino nucleic acid) [32], GNA (glycol nucleic acid) [33], and LNA [34] (Figure 2).

In order to evaluate the feasibility of using XNAs for heredity and evolution, a replication system should be developed. Non-enzymatic replication of nucleic acids has been studied to understand the chemical origin of genetic materials [35,36]. It was found that chemical replication suffers with insufficient efficiency, low fidelity, and limited length of polymerization. In nature, replication is catalyzed by DNA polymerase with high efficiency and fidelity. Due to the structural difference between XNAs and DNA/RNA, normally, XNAs are not good substrates for natural DNA polymerases. Even though genetic information is stored in XNA sequences, it is still invisible to the natural system. With the help of directed evolution, specific XNA polymerases were obtained to transfer the genetic information back and forth between XNA and DNA with reasonable fidelity and efficiency [17,18]. Using a compartmentalized self-tagging (CST) selection strategy, specific XNA polymerases that can synthesize HNA/CeNA from DNA templates were obtained by Holliger and coworkers [21]. XNA replication systems with high fidelity were built for six XNAs (HNA, CeNA, LNA, ANA, FANA, and TNA). The replications between FANA and FANA, CeNA and CeNA, or HNA and CeNA were also realized based on these engineered polymerases.

Next, the capacity of XNAs for Darwinian evolution was evaluated by in vitro selection of functional aptamers and catalysts. Aptamers are single-stranded oligonucleotides that fold into complex 3-D structures and bind to specific targets, and are commonly produced by systematic evolution of ligands by exponential enrichment (SELEX), also referred to as in vitro selection or in vitro evolution. To select XNA aptamers, the XNA library used in SELEX has to be amplified by XNA polymerases, and selected XNAs should be able to be transcribed into cDNA. An HNA aptamer binding to the HIV TAR RNA motif was obtained with the dissociation constant (KD) between 28 and 67 nM by using the above-mentioned HNA replication system [21]. Similarly, FANA aptamers that bind to HIV-1 integrase or reverse transcriptase (RT) were selected with picomolar affinities [37,38]. Computational design and sampling and pooling of beneficial mutations were utilized by Chaput’s group to generate TNA polymerase Kod-RI with high efficiency [39,40]. With this enzyme, a biologically stable TNA aptamer was discovered with a high binding affinity to HIV RT [41]. Starting from random XNA oligomer pools, functional XNAs (ANA, FANA, HNA, and CeNA) with trans-RNA endonuclease and ligase activities (XNAzymes) were obtained by in vitro selection [42]. Recently, a general RNA-cleaving FANA enzyme (FANAzyme) that functions with enhanced activity was evolved in the laboratory by Chaput’s group [43].

In summary, like DNA/RNA, these sugar-modified XNAs are also capable of undergoing Darwinian evolution to acquire specific target binding affinities or catalytic activities [21,41,42,43,44]. These results reveal that some XNAs, which are capable of heredity and evolution, can be used as alternative genetic materials in vitro.

The in vivo implementation of sugar-modified XNA, such as HNA and CeNA, into living systems was carried out by Herdewijn and coworkers. HNA nucleoside analogues were initially developed as potential antiviral agents due to their potent activities against DNA viruses [45]. Later on, HNA oligonucleotides were prepared and found to be chemically and enzymatically stable nucleic acids that hybridized with DNA, RNA, and itself based on its structural characteristics [28]. CeNA is composed of carbocyclic nucleotides that contain a cyclohexenyl sugar moiety with a C=C double bond, which makes the cyclohexene system more flexible than furanose ring. Interestingly, it is reported that similar conformation changes between CeNA and natural DNA were observed, indicating the possibility of using CeNA as a biocompatible polymer [30].

**Figure 3:**
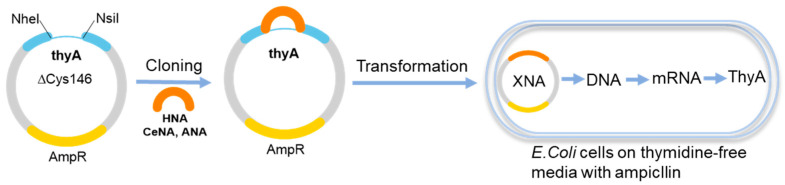
Genetic information transfer from XNAs to DNAs in vivo.

Up to six continuous HNA or CeNA nucleotides in the template were read by the *E. coli* cell machinery to give ThyA-produced colonies [46]. The *thyA* gene encoded thymidylate synthase, which is necessary for *E. coli* cells to grow in thymidine-deficient medium. To introduce HNA into the plasmid, restriction enzymes NheI and NsiI were utilized to digest the *thyA* gene, and six codons around the cysteine at position 146 were deleted to create a gap for the ligation of XNAs (Figure 3). After ligation, the plasmids were transformed into a *thyA*-deletion strain to test their biological activity. The results showed that HNA served as a short template to produce the active *thyA* gene and restore the function of thymidylate synthase. The relatively poor acceptance of HNA for the endogenous replication machinery led to a decreased number of thyA+ colonies. Even though T and G were replaced by uracil and hypoxanthine in the these XNAs, ANA and HNA still succeeded in restoring the functional *thyA* gene and generating survival colonies. Similar results were obtained when in vivo genetic selection screening was performed with CeNA and ANA (Figure 3). Unfortunately, mosaic template containing GNA did not yield any of the thyA colonies even though one codon was substituted by GNA.

**Figure 4:**
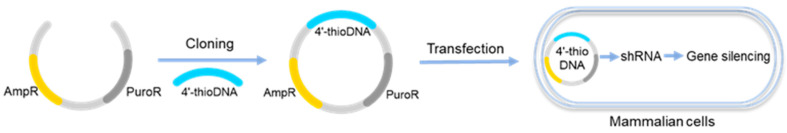
4′-ThioDNA with gene-silencing activity in mammalian cells.

Although genetic information encoded in XNAs can be transliterated to DNAs, it is difficult for these XNAs to directly function as a template for transcription. However, for 4′-thioDNA, a natural-like XNA in which the 4′-oxygen atom is substituted by a sulfur atom, the direct transcription can be performed in human cells. As reported, 4′-thioDNA is resistant against nuclease and hybridizes like RNA molecules [47]. 4′-ThioDNA containing 4′-thio-dT and 4′-thio-dC was amplified by DNA polymerase from *Pyrococcus kodakaraensis* (KOD dash DNA polymerase) and used as template for transcription both in vitro and in vivo to produce RNA with gene-silencing activity (Figure 4) [48]. Further exploration of the transcription was carried out by using 4′-thioDNA templates with 4′-thioA and 4′-thioG nucleotides to afford luciferase in murine cells, showing that 4′-thioDNA is a potential candidate for the development of a synthetic genetic system [49].

Chemical alteration of the ribofuranose ring induces conformation changes that consequently lead to a lower level of acceptance of synthetic nucleotides by natural DNA/RNA polymerases. Directed evolution affords engineered polymerases that can transfer the genetic information to promote the application of XNAs in living cells [19]. Significant achievements have been achieved for the development of engineered polymerases that can transfer genetic information between XNAs and DNA to date. The next challenge is to develop XNA-templated XNA polymerases for XNA replication. Engineered RNA polymerase that can transcribe XNAs into RNAs is needed for expanding the central dogma with XNAs. The discovery of these new enzymes may allow us to create artificial life forms based on XNA genetic systems.

### 2.2. Phosphate-Modified XNAs

**Figure 5:**
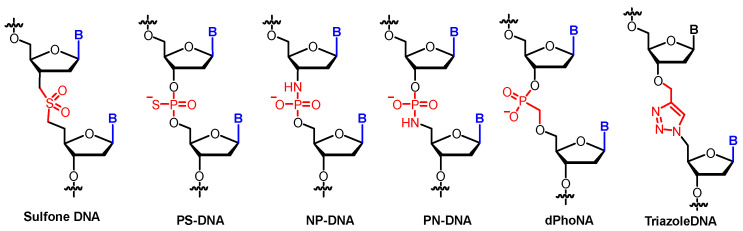
Examples of phosphate linkage-modified XNAs.

The classic phosphate diester linkages are substituted by different functional groups in sulfone-DNA [50], PS-DNA [51], PN-DNA [52], NP-DNA [53], triazole-DNA [54], and dPhoNA [55] (Figure 5).

In natural DNA/RNA, phosphate diester is the linker between nucleosides and is negatively charged at physiological pH. It is demonstrated that the repeating negative charges retain DNA/RNA within lipid membranes and stabilize the diester linkages against hydrolysis [56]. Investigations on an uncharged DNA analogue in which the phosphate diester was replaced by a dimethylenesulfone group [50] showed that charged phosphate linkage is also important for base-pairing and Darwinian evolution [57]. Even though the short oligomer of sulfone-DNA exhibits a similar geometry to natural DNA with G:C and C:G pairing [58], self-aggregation was observed when the oligomers were long enough. Single nucleobase alternation leads to significant changes in the solubility, aggregation, and chemical reactivities of sulfone-DNA, suggesting that it is not a suitable genetic polymer in water.

PS-DNA, a modified XNA in which the non-bridging oxygen of the phosphate diester is replaced by a sulphur atom, was synthesized, aiming to develop antiviral agents against HIV in the 1980s [59]. The phosphorothioate motif shares a similar structure with the commonly seen phosphate diester linkage and offers considerable advantages, such as enhanced resistance to nucleases [60]. It is also reported that naturally occurring phosphorothioation was discovered in bacterial DNA and played an important role in many cellular processes [60]. The replacement of oxygen by sulphur takes place under the catalysis of the gene products of the *dndABCDE* cluster. PS-DNA, together with DndFGH, constitutes a defense system that can distinguish and attack non-PS-modified foreign DNA [60,61,62]. PS-DNA is an alternative genetic material that can be potentially used for the construction of artificial life forms.

DNA with N3′→P5′phosphoramidate linkages (NP-DNA) was first developed by Gryaznov and co-workers for therapeutic applications [53,63]. Although capable of forming stable duplexes with both RNA/DNA strands, NP-DNA is functionally more similar to RNA because its structure closely resembles the classical A-form RNA structure. It is used as a model to study the nonenzymatic template-directed replication, which may provide insight into the chemical replication of genetic materials [64]. NP-DNA can be synthesized from RNA template and extended up to 25 nucleotides inside a model protocell [65]. Enzymatic replication or reverse transcription of NP-DNA were performed by Szostak and coworkers, showing that NP-DNA might be a good candidate for the construction of synthetic life [52,66]

Recently, Herdewijn’s group reported the in vivo study of a synthetic genetic polymer bearing the P3′→N5′ phosphoramidate linkages, which is denoted as PN-DNA [67]. Compared to DNA, PN-DNA is more stable under basic conditions and more acid-labile, such that the phosphoramidate linkage can be cleaved under acid conditions [68]. Enzymatical synthesis of PN-DNA was successfully achieved by employing Taq DNA polymerase together with KF (Klenow fragment) and Vent (*exo*^−^) polymerases. Multiple NH-dCTPs were incorporated into the R67DHFR gene sequence to give modified plasmids with trimethoprim resistance, which was then transformed into *E. coli* cells on trimethoprim-containing medium. With sufficient accuracy, the in vivo transliteration of PN-DNA to natural DNA was achieved, thus providing viable colonies with antibiotic resistance.

dPhoNA represents a family of nucleic acids that contain a phosphonate linkage rather than the natural phosphate diester. Phosphonate is an isostere of phosphate diester that contains a stable P-C bond, which is resistant against chemical and enzymatical degradation. Therminator polymerase is demonstrated as a catalyst for the condensation of the phosphonate derivatives of adenine to afford dPhoNA with enhanced stability against nucleolytic degradation [69]. However, the polymerases are not able to catalyze the synthesis of a sequence longer than four different nucleotide phosphonates [70]. Directed evolution may provide better polymerases with improved recognition functions for the development of dPhoNA-based genetic systems.

**Figure 6:**
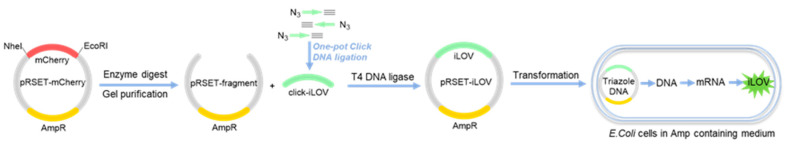
Transcription and translation of triazoleDNA in vivo.

Another interesting and successful example of phosphate-modified XNAs is triazoleDNA, which comprises a triazole ring between nucleotides instead of natural phosphate diesters. As presented in Figure 6, treatment of the pRSER-mCherry plasmid with NdeI and EcoRI leads to the cleavage of the *mCherry* gene, furnishing pRSER fragments after gel purification. The azide-alkyne click reaction was employed for the assembling of the *iLOV* gene, which was then ligated to the pRSER plasmid in the presence of T4 DNA ligase. The biocompatibility of triazole linkers in the *iLOV* gene was examined by in vivo replication and transcription in *E. coli* cells to produce a green fluorescent protein successfully [71]. As the first example of a non-natural linker being functional in human cells, triazoleDNA was directly transcribed into mRNA and encodes the fluorescent protein [72]. Notably, the overall physical and chemical properties of DNA were maintained after the insertion of a limited number of triazole linkers into DNA.

### 2.3. Sugar- and Phosphate-Modified XNAs

**Figure 7:**
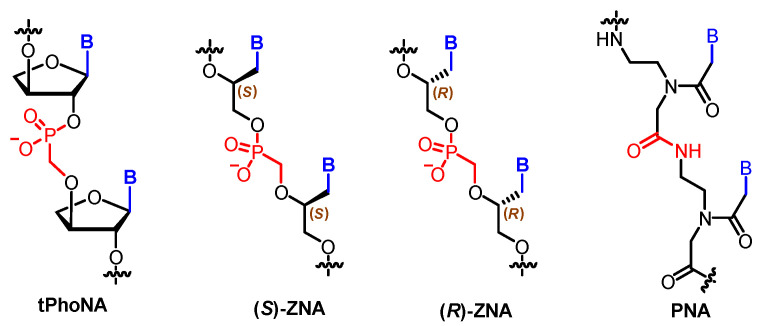
Structures of tPhoNA, ZNA, and PNA.

Both the sugar moieties and the phosphate are chemically modified in some nucleoside analogues. The combination of phosphonate linkages and artificial sugar rings gave rise to the discovery of two orthogonal XNAs: tPhoNA (3′–2′ phosphonomethyl-threosyl nucleic acid) [73] and ZNA (an XNA analogue with an acyclic methylphosphonate backbone) [74]. The former contains a threose ring while the latter one bears an acyclic backbone (Figure 7). Initially, the repeating nucleoside units of both tPhoNA and ZNA are developed for antiviral drug discovery [75,76]. tPhoNA successfully served as a xenobiotic genetic material in vitro in the presence of engineered polymerases. Further in vivo studies showed that tPhoNA was not a good substrate for *E. coli* DNA polymerases, indicating that tPhoNA was not accessible to the natural system. Based on the obtained results, an orthogonal genetic system could be built with tPhoNA and laboratory-evolved tPhoNA polymerases.

Due to the existence of a chiral carbon center in the backbone, ZNA is obtained as two enantiomers, (*S*)-and (*R*)-ZNA. (*S*)-ZNA strongly hybridizes with itself and interacts weakly with the complementary single-stranded DNA, showing that ZNA is chemically orthogonal. Biologically, the diphosphates of (*S*)-ZNA monomers can be recognized as substrates by *E. coli* polymerase I and the algal nucleotide transporter. Successful in vivo transliteration of ZNA to DNA was conducted inside *E. coli*, which proved that ZNA can be potentially used for genetic information propagation.

Protein, which is composed of amino acid residues linked by amide bonds, is another biological polymer in living organisms other than DNA/RNA. Whether polyamide linkers could be used instead of phosphate diesters in DNAs has been investigated towards the development of alternative genetic materials. Invented by Nielsen, Egholm, and their collaborators, peptide nucleic acid (PNA) is a neutral oligomer in which the whole deoxyribose phosphodiester backbone is replaced by N-(2-aminoethyl)glycine [77] (Figure 7). It forms very stable duplex structures with complementary DNA, RNA, or PNA. PNA has been used in the areas of gene therapy, genetic diagnostics, and nanotechnology [78].

As a molecule that lies between protein and DNA, PNA was studied to test the origin of life since it might be synthesized and used in the early Earth. The synthesis of PNA building blocks was achieved by Nielson under simulated prebiotic conditions [79]. Orgel and coworker demonstrated that the genetic information transfer between PNA and RNA was successful [80]. The description of the PNA self-replicating system and non-templated PNA replication prompted the speculation that PNA may be a prebiotic genetic material [81,82]. According to Benner, the repeating charge in DNA might be a universal feature of genetic molecules in water, including those in alien life throughout the cosmos [57]. Without negative charges, the self-aggregation and precipitation of PNA in water were observed [83]. The possibility of using PNA as genetic materials or prebiotic genetic materials is still unclear, thus further investigation is required.

### 2.4. Base-Modified XNAs

Genetic information is stored in nucleic acid sequences and transmitted following Watson–Crick base-pairing rules during DNA replication and transcription. In nature, some nucleobases are damaged by oxidation, depurination, deamination, or other mutagens, leading to decreased accuracy of information transfer [84,85]. Base-modified XNAs with improved properties are considered and used instead of natural DNAs.

The functionalities of DNAs have been widely expanded by chemical manipulation of nucleobases over the past few decades. These unnatural nucleobases are used in SELEX for the development of modified aptamers and deoxyribozymes with desired functions [86]. For example, selections from DNA pools containing 2′-deoxyuridine derivatives with hydrophobic groups at the 5-position of the nucleobase generate aptamers that have more accessible epitopes on protein targets and broader scope of applications in diagnostics and therapeutics [87]. In addition, SELEX and related in vitro selections are utilized to obtain DNAzymes, which are highly specific catalytic DNAs bearing modified bases [88]. Some RNA-cleaving DNAzymes having protein-like side chains attached to the bases were found to be active in the absence of divalent metal ions [89] despite the fact that most of the selected DNAzymes require multivalent metal cations for decent activities.

**Figure 8:**
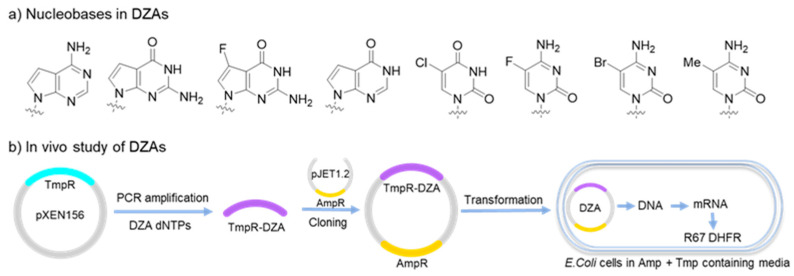
DZAs as alternative genetic materials. (**a**) Non-canonical nucleobases used in DZAs; (**b**) DZAs used as genetic template in bacterial cells.

DZA, described as ‘a fully morphed DNA containing all four non-conical nucleotides’, was developed by Herdewijn’s group. Unnatural bases used in DZAs share similar skeletons with their natural counterparts (Figure 8a), including 5-chloro-2′-deoxyuridine (5ClU), 5-methyl-2′-deoxycytidine (5MeC), 5-fluoro-2′-deoxycytidine (5FC), 7-deaza-2′-deoxyadenosine (7dA), 7-deaza-2′-deoxyguanosine (7dG), 7-fluoro-7-deaza-2′-deoxyguanosine (7FG), and 2′-deoxyinosine (dI). Taq or Vent (*exo*^−^) DNA polymerases served as catalyst for the PCR amplification of DZAs. DZA fragments efficiently block the restriction sites from enzymatic cleavage, representing a better property as alternative genetic materials. In vivo studies were performed to examine the biological functions of DZAs. A pXEN156 plasmid was employed as parent template to synthesize DZA-containing genes by PCR amplification, followed by ligation to a pJET1.2 plasmid with ampicillin resistance (AmpR) by T4 DNA ligase. The modified plasmid was then transformed into *E. coli* cells cultured in media with Amp and trimethoprim (Tmp). Transliteration of DZAs was successfully achieved in *E. coli* cells to generate the R67DHFR protein, which is resistant to Tmp (Figure 8b) [90,91]. The results suggest that DZAs are biocompatible genetic materials with new functionality and can be used for the construction of synthetic life forms. Additionally, such DZA libraries can be potentially used for the selection of functional aptamers and DNAzymes.

**Figure 9:**
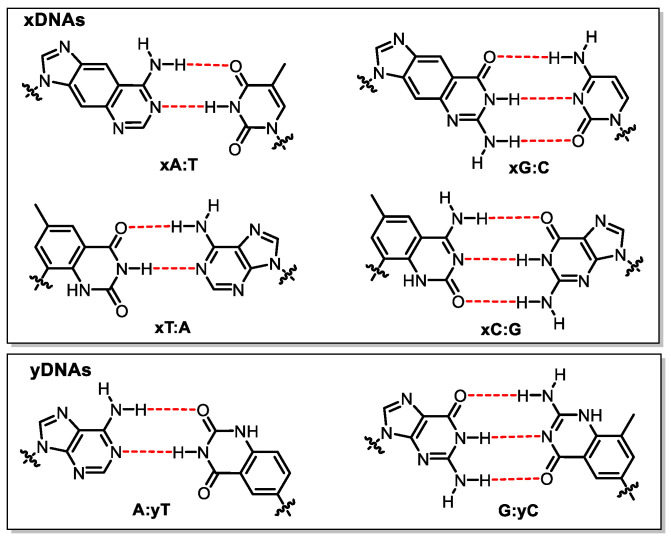
Structures of sized-expanded bases in xDNA and yDNA.

Modified bases that consist of fused aromatic rings for the construction of synthetic genetic system were synthesized and evaluated by Kool’s group. Embedded with size-expanded artificial bases (xDNA and yDNA, Figure 9), these DNAs are fluorescent and display high stacking affinity compared to natural DNA [92]. They were utilized to build a synthetic genetic set through cloning and expression in *E. coli* cells [93,94]. The pGFPuv plasmid was digested by the restriction enzymes BsrGI and MluI followed by the removal of 5′-phosphate by antarctic phosphatase to afford DNA fragments ready for the ligation of xDNA segments. After ligation under T4 DNA ligase catalysis, the xDNA-containing plasmid was transformed into *E. coli* to yield green fluorescent protein successfully. It was demonstrated that a maximum of four consecutive modified base pairs can be read accurately by cellular enzymes in *E. coli* to generate native DNA and express functional proteins [93]. Further explorations of sized-expanded yDNAs were executed by testing their ability to store and transfer genetic information in vitro and in vivo. Primer extension assays were performed with KF exo^−^ and the thermostable DNA polymerase Vent exo^−^, showing that the enzymes incorporated the natural nucleotides’ opposite yDNA bases correctly, but the selectivity was unsatisfactory (T-yT and T-yC mispairing was observed). Plasmids with one or two yDNA bases were evaluated in vivo, leading to the generation of a functional protein [94].

Complete replacement of natural nucleobases with non-canonical ones in the genomes of living organisms is challenging. Peter G. Schultz and coworkers reported that engineered *E. coli* cells were obtained by replacing thymidine with 5-hydroxymethyluridine (5hmU) [13], or the replacement of cytidine with 5-hydroxymethylcytosine (5hmC) [14]. For 5hmU incorporation, the metabolic pathway of pyrimidine nucleotides in *E. coli* was engineered to mimic the metabolic approach used by SPO1 bacillus phage. A series of biochemical reactions that converted deoxyuridine monophosphate (dUMP) into 5hmdUTP took place in engineered *E. coli* under the catalysis of cellular enzymes, including hydroxymethylase, mononucleotide kinase, and nucleotide diphosphate kinase. The synthesis of TTP was inhibited by the disruption of the *thyA*, *deoA*, and *trmA* genes of *E. coli* as TTP is the competitor of 5hmdUTP during DNA replication. As a result, 5hmdUTP was successfully incorporated into the *E. coli* DNA genome and approximately 75% of the thymidine was replaced by 5hmU [13]. Similarly, the replacement of natural dC with 5hmC residues was studied. The biosynthetic pathway of *E. coli* was manipulated using bacteriophage T4 genes to incorporate 5hmC into the genome. Having these modified genes, the intracellular synthesis of 5hmdCTP was successfully achieved, offering 5hmdCTP as competitive substrates with dCTP for DNA synthesis. The results showed that about 63% of 2′-deoxycytidine was displaced by 5hmC in the *E. coli* genome [14]. Further experiments may be required to investigate the key physiological regulators that limit entire genomic replacement.

Surprisingly, mutated *E. coli* strains containing chimeric DNA–RNA sequences (40–50% ribonucleotide content) in their genomic DNA were observed during the exploration to improve 5hmC content [95]. It has been described that DNA polymerases can incorporate ribonucleotides and DNA–RNA chimeras are eligible for transcription. Recent studies postulate that homogeneous DNA genomes may have evolved from heterogeneous chimeric RNA–DNA templates [96]. The detection of this DNA–RNA chimera may provide further insights into the origin of life [95].

Substitution of thymidine by bromodeoxyuridine (BrdU) in the DNA of the *E. coli* strain TAU-bar [97] or a hamster cell line [98] was performed decades ago. Even though the van der Waals radii of the bromine atom and methyl group are almost the same, the higher electronegativity of bromine affects the electron distribution of the aromatic ring, which may result in mispairing in DNA owing to a keto-enol tautomerism [97]. The replacement was achieved smoothly in that BrdU was quantitatively incorporated into *E. coli* DNA. As demonstrated by Davidson, the level of BrdU substitution in the DNA of the hamster cell line was higher than 99.8%, and possibly 100%. However, in both cases, the presence of high levels of BrdU in the genomic DNA raised some problems, such as a similar phenomenon to thymineless death and mutagenesis caused by base transitions.

Recently, investigations on the evolution of genomic DNA of *E. coli* strains with 5-chlorouracil (5-ClU) were conducted by Rupert Mutzel and coworkers, showing that 98.4% of thymine was displaced [99]. Despite the presence of chlorine in various natural products, it is not found in any natural nucleobase. The reasons for choosing 5-ClU as a substitute for thymine include (i) the structure of 5-ClU closely resembles thymine, which allows it to form a stable base pair with adenine; (ii) it can be converted into chlorodeoxyuridine nucleotides by a panel of cellular enzymes; and (iii) it is chemically more stable than fluoro, bromo, and iodo analogues, which enables it to avoid being reduced to uracil in the cytoplasm and ambiguous pairing caused by tautomerism [99]. The bacterial strain THY1 used for the transliteration of 5-chlorouracil was prepared by reconstruction of a wild-type *E. coli* strain MG1655, including deletion of the *thyA* and *udp* genes and the *deoCABD* operon, and addition of a plasmid containing the *Lactobacillus leichmannii* gene *ntd*. After cultivation and evolution for 25 weeks, the modified strains were able to grow with chlorouracil and the thymidine residual fraction was below 2%. As a result, 1502 genetic mutations were observed in this artificially constructed strain due to the genomic changes, revealing the difficulty of replacing DNA with XNA in the bacterial genome.

### 2.5. Unnatural Base Pairs (UBPs)

**Figure 10:**
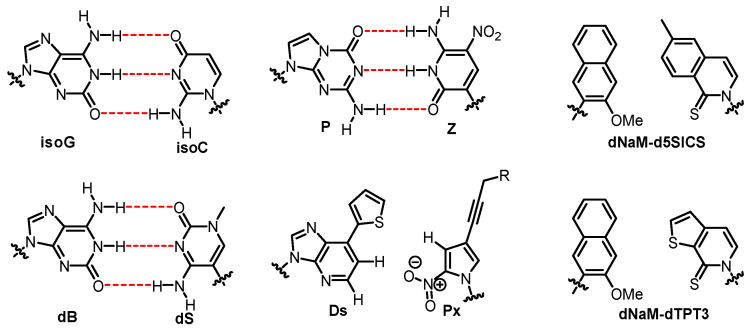
Structures of UBPs.

Although the nucleobase, sugar, or backbone moieties have been reconstructed in the above mentioned DZAs or XNAs, Watson–Crick base pairing geometry is still adopted and no new genetic codon is created. In contrast, insertion of a third base pair (UBP) other than A:T and G:C into DNA strands greatly expands the genetic code, offering an opportunity to create more life forms that do not exist in nature [100,101,102]. Numerous unnatural base pairs (UBPs), also known as artificially expanded genetic alphabets, have been developed ever since the first UBP (isoG: isoC) was synthesized and incorporated successfully into DNA/RNA by Steven Benner in 1989 [103]. Other UBPs based on altered hydrogen bonding were created, such as dB:dS and P:Z base pairs (Figure 10) [104,105], and subsequently used for the construction of a genetic system containing eight letters. Compared with the natural genetic system, eight-letter DNA and RNA possess an enhanced ability to store data and may provide insights into the search for extraterrestrial life [105].

Despite the fact that hydrogen bonding is very important for the formation and stability of the DNA structure, it is not the only driving force. It has been addressed that base stacking, the neighboring interaction between aromatic π-electron systems of the bases, is another factor that affects the stability of base pairing. In 1995, the first synthesis and in vitro evaluation of nonpolar base pairs that pair through shape complementary were presented by Kool’s group [106], providing evidence that a hydrogen bond is not absolutely required for the synthesis of DNA. The corresponding hydrophobic base pairs were incorporated into oligonucleotides to form stable self-pairing patterns, raising the possibility of adopting hydrophobic base pairs to expand genetic alphabet. Some hydrophobic UBPs are comprised of heterocycles with totally different structures, for instance, Ds:Px [107], dNaM:d5SICS [108], and dNaM:dTPT3 [109] base pairs (Figure 10). The Ds:Px base pair is a representative hydrophobic base pair developed by Hirao’s group. Initially, the research of Hirao’s group focused on the development of hydrogen-bonded UBPs. However, they started the investigation on hydrophobic UBPs because the selectivity of their hydrogen-bonded UBPs is insufficient during replication, and small hydrophilic bases are not good substrates for polymerases [110]. It has been illustrated that these UBPs are biologically functional as third base pairs during the replication, transcription, and/or translation process in vitro [100,101,102].

The application of UBPs in the field of drug therapy and synthetic biology has been investigated. Developed by Benner and coworkers, aptamers containing a third base pair (P:Z, Figure 10) were applied to recognize HepG2 liver cancer cells [104]. Aptamers targeting the human vascular endothelial growth factor (VEGF165) with pM affinity were selected from DNA pools containing Ds nucleotides [111]. dNaM:d5SICS and dNaM:dTPT3 base pairs are successful UBPs created by Romesberg’s group that have been used for the creation of semi-synthetic organisms.

In synthetic biology, to create artificial life with UBPs, challenges that have to be overcome include the transport of unnatural triphosphates into cells, faithful replication of UBP, and maintenance of UBP-containing DNAs during cell division. In 2014, Romesberg’s group succeeded in constructing semi-synthetic strains of *E. coli*, in which a genome that contains a hydrophobic base pair can be stably propagated [108]. The algal transporter PtNTT2 was found to be an effective transporter for the importing of d5SICSTP and dNaMTP into *E. coli* and pol I polymerase was believed to replicate a plasmid containing UBPs faithfully and efficiently. This UBP is fairly stable against DNA repair pathways, affording the first semi-synthetic organism (SSO) with an expanded genetic alphabet.

**Figure 11:**
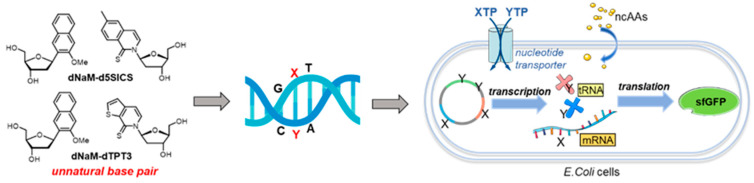
Construction of semi-synthetic organisms with UBPs.

Based on the above-mentioned results, further investigations on the in vivo transcription and translation of UBPs were performed [109]. A new base pair dNaM-dTPT3 was prepared and then transcribed into mRNA and tRNA to form unnatural codons and the corresponding unnatural anticodons, respectively (Figure 11). The uptake and incorporation of non-canonical amino acids (ncAAs) were realized to yield a green fluorescent protein, which means the successful creation of SSO with new functions. Besides, DNAs that lost the UBP can be eliminated by CRISPR (clustered regularly interspaced short palindromic repeats)-Cas9 (associated protein 9) technology to further promote the health of synthetic organisms, allowing more attractive prospects in the development of artificial life with UBPs [112]. Lately, it was described that nine unnatural codons were identified with the ability to produce unnatural protein through incorporation of an encoded ncAA. Three of the codons, which are orthogonal and can be decoded, were utilized to construct the first SSO with 67 codons, allowing the development of proteins and new life forms with novel functions [113].

## 3. Conclusions and Outlook

A variety of unnatural nucleic acids have been synthesized by replacing natural bases, sugars, and phosphate linkages with artificial structures to investigate their potential as alternative genetic materials. The key chemical and structural parameters for genetic information storage, heredity, and evolution have been elucidated. Engineered polymerases promoted the application of XNAs as alternative genetic materials in vitro. However, it is still challenging to use XNAs instead of DNAs in living cells. In order to know whether this idea is feasible, exploration of xenobiotic life with XNA as genetic material is necessary.

Initial studies on the in vivo implementation of XNAs have been performed, and it is demonstrated that plasmids containing sugar, backbone, or nucleobase-modified XNAs can be read by the cellular machinery to generate natural DNA with correct genetic information. The central dogma is expanded through the insertion of UBPs, resulting in the successful construction of semi-synthetic organisms with an extended genome. Current achievements are encouraging for the integration of unnatural genetic materials into the living system to create synthetic life forms [114,115,116]. The construction of synthetic life forms is helpful to reveal the fundamental principles of living systems and enlighten our understanding of current life on Earth.

However, as alternative genetic materials, XNAs should be able to replace DNA in living organisms with complete genome substitution. Up to now, only few examples of artificial life whose whole genome was reconstructed is reported, in which the natural base thymine is replaced by 5-postion-modified uracil after chemical evolution. As a consequence of this replacement in DNA, significant mutations in the resulting bacterial genome were detected. Although some promising results have been obtained in this research area, the construction of XNA-based synthetic life is still in its infancy, thus more effort is needed to achieve this goal.
